# Primary Omental Torsion in an Old Woman: Imaging Techniques Can Prevent Unnecessary Surgical Interventions

**DOI:** 10.1155/2011/541324

**Published:** 2011-06-16

**Authors:** Mohhamad-Hadi Saeed Modaghegh, Reza Jafarzadeh

**Affiliations:** ^1^Mashhad Vascular and Endovascular Research Center, Mashhad University of Medical Sciences (MUMS), Mashhad, Iran; ^2^General practitioner, Mashhad Vascular and Endovascular Research Center, University of Medical Sciences (MUMS), Mashhad, Iran

## Abstract

Torsion and/or infarction of the greater omentum are rare but well-recognized clinical situations which present as an acute abdomen. The etiology is unknown and speculative. In most cases, the pathology is right sided and clinical presentation consists of an acute or subacute flank pain with mild peritonism usually evoking appendicitis or cholecystitis. Nevertheless, knowledge concerning these two problems can help the surgeon in proper diagnosis and treatment. Since the first report on primary torsion by Eitel in 1899, a few hundred more have been reported and some collective reviews published to date. Recently, ultra sonography and computed tomography have proved to provide sufficiently typical, consistent, and well-recognizable features to avoid unnecessary surgery. In this study, we will present a case diagnosed as primary omental torsion based on computed tomography, which underwent successful conservative management.

## 1. Background and Aims

Vascular accidents of the omentum including torsion, infarction, and hemorrhage are all rare causes of the acute abdomen. They are usually misdiagnosed in the majority of cases as acute appendicitis, followed by acute cholecystitis. Other differential diagnoses are perforated peptic ulcer, appendicular abscess, pancreatitis, torsion of ovarian cyst, and diverticulitis [1, 2]. Therefore it is important that responsible physicians be aware of this entity. It is recommended that in all negative exploratory laparotomies the greater omentum should be evaluated routinely to avoid missing an instance of infarcted omentum. 

 Spontaneous detorsion is an expected possibility [3, 4]. This may be a cause of chronic abdominal pain with obscure origin [5]. According to this phenomenon, nonsurgical treatment has also been proposed [6, 7]. 

 In this study a case of primary omental torsion, conservatively managed is presented with a review of literature, indicating that unnecesseary surgery can be successfully avoided. 

## 2. Case Report

 A 74-year-old woman was admitted to hospital with a 4-days history of abdominal pain. The pain started suddenly in the upper abdomen, increased in severity and localized in the epigaster and right upper quadrant. The pain was constant, and associated with nausea, vomiting and back radiation. There was a previous history of diabetes mellitus (type II), ischemic heart disease, and chronic hypertension. On examination, blood pressure was 160/95 mmHg and other vital signs were normal. Abdominal examination revealed generalized guarding and tenderness with major severity and rebound tenderness in the right upper quadrant. Bowel sounds were hypoactive, and there was no abdominal distension. A tender and ill-defined fullness was palpable. Total white blood cell count was 9500/mm^3^. 

 Ultrasound (US) examination was performed which revealed a heterogenous echogenic mass in the upper abdomen. No caecal and appendiceal abnormality was identified. Kidneys and hepatobiliary system were also normal. 

**Figure 1:**
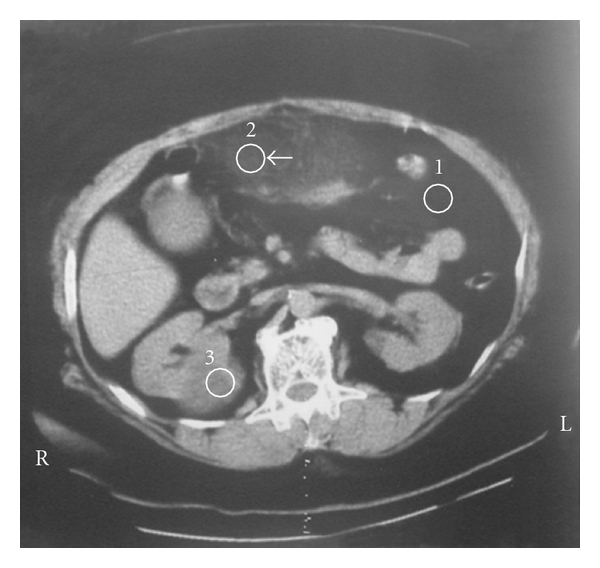
Axial CT view illustrating the presence of an inflammatory focal fatty mass just under the anterior abdominal wall.

 As the patient's abdominal tenderness persisted the next day, a computed tomography (CT) scan was requested which demonstrated a 12 × 7 cm amorph and solid mass at the site of tenderness in the upper abdomen. It was adherent to the anterior wall of abdomen at the midline (Figure 1). There was no evidence of pancreatic disease. The heterogenic mass seen on CT scan was corresponding to a focal, well-demarcated area of greater omentum. 

 The patient was managed conservatively with oral analgesic and antibiotics. The pain gradually resolved in 2 days and she was discharged after 9 days of hospitalization. Upon review in the outpatient clinic at 1 week, 1 month and 6 months after discharge, the patient remained well. She did not consent to perform CT scan for follow up. 

## 3. Discussion

Acute conditions of the omentum including torsion, infarction and hemorrhage are all rare but well-recognized conditions. Omental torsion was first described in the literature by de-Marchetti in 1858 and primary torsion of the omentum by Eitel in 1899 [8]. Until 1993 less than 350 cases have been published in the literature [9]. Also we have reported 7 similar cases published in 1997 [8]. 

 Torsion of the omentum is usually segmental and may be idiopathic or secondary to intra-abdominal inflammatory foci, adhesions and internal hernias [7]. The pathogenesis is poorly understood. Primary torsion is usually related to omental anomalies. Secondary torsion usually occurs in association with intra-abdominal pathology such as hernia, tumor, cyst or adhesions. Predisposing factors for primary or secondary torsion are similar mainly a sudden increase in intra-abdominal pressure [10]. In our patient, no precipitating factor or predisposing intra-abdominal pathology was present. 

There are several clinical peculiarities of primary segmental omental infarction that have been established. Patients usually present with acute abdominal pain without other gastrointestinal symptoms. They are usually constitutionally well, without fever or leucocytosis. Focal tenderness with varying degrees of peritonism is found on examination. Given that segmental infarction is usually right sided, appendicitis is the usual presumed diagnosis or, rarely, cholecystitis. Since it is such a rare condition, the diagnosis is usually made at surgery, but is occasionally discovered preoperatively during cross-sectional imaging. 

 In recent years, the correct preoperative diagnosis of omental torsion via US or CT scan has increasingly been reported in the literature [9, 11]. US typically demonstrates a characteristic pattern of a noncompressible hyper echoic ovoid mass located exactly under the site of maximum tenderness directly under the abdominal wall. The mass is frequently more or less circumscribed by a hypoechoic line corresponding to the inflammatory peritoneum. However, the diagnosis may be missed on US, as in our case, because of a lack of awareness. On CT scan, an oval, round-shaped mass of slightly hyperattenuating fat interspersed with water-dense streaks and hyperdense spots indicative of edema and hemorrhage closely related to the ascending on descending colon is characteristic [9, 12]. It is essential to remember that a definitive diagnosis of torsion or infarction can only be made radiologically if the above-mentioned features are seen in conjunction with the absence of any specific imaging findings of other acute abdominal conditions such as appendicitis, diverticulitis or cholecystitis [9].

 In this case, regarding the typical clinical manifestations, computed tomography was performed which demonstrated an amorph and solid mass at the site of tenderness in the upper abdomen, adherent to the anterior wall. The heterogenic mass seen on CT scan was corresponded to a focal, well-demarcated area of greater omentum. These findings suggest that clinical symptoms can clearly lead to pathologic diagnosis.

 Previously, omental torsion was almost always managed operatively as this condition was usually found unexpectedly during surgery [9]. However, with the increased availability and use of cross-sectional imaging such as US and CT scan, increasing numbers of these cases have been successfully diagnosed preoperatively and hence treated conservatively [13, 14]. With the conservative management, the symptoms usually resolve gradually over 1 or 2 weeks with oral analgesics. Antibiotics, although administered prophylactically in our patient, are not essential in the treatment of this condition [12]. It is important to note that omental torsion is not always benign and self-limiting. Complications as a result of conservative treatment such as adhesions causing intestinal obstruction, septic shock, and intra-abdominal abscess have been reported in the literature [9]. Hence, patients with omental torsion managed expectantly should be closely monitored and if discharged given appropriate medical advice. Surgical intervention, preferably via laparoscopy, is mandatory if their clinical condition deteriorates. However, despite the increasing reports in the literature of successful conservative management of this unusual condition, some authors still advocate surgical treatment, citing its low operative morbidity and the potentially quicker resolution of symptoms. As mentioned above, conservative management is safe especially when symptoms resolve in few days and under accurate management. 

 In summary, we have reported a case of omental torsion diagnosed by CT scan and successfully managed nonoperatively. These imaging findings are important to recognize because unnecessary surgery may be avoided when the patient's clinical condition remains stable. However, if the diagnosis is uncertain, diagnostic laparoscopy or laparotomy should be performed to confirm the diagnosis and exclude other sinister causes.
